# Commentary: Focus on the Gut–Kidney Axis in Health and Disease

**DOI:** 10.3389/fmed.2021.669561

**Published:** 2021-05-03

**Authors:** Yulu Li, Bin Zhu, Chun Yang, Liying Miao

**Affiliations:** ^1^Department of Nephrology, The Third Affiliated Hospital of Soochow University, Changzhou, China; ^2^Department of Critical Care Medicine, The Third Affiliated Hospital of Soochow University, Changzhou, China; ^3^Department of Anesthesiology and Perioperative Medicine, The First Affiliated Hospital of Nanjing Medical University, Nanjing, China

**Keywords:** chronic kidney diseases, gut microbiota, gut-kidney axis, hemodialysis, bacterial translation

We read with great interest the article by Stavropoulou et al. (1), in which the authors aimed to investigate the interactions between human gut microbiome and kidney diseases. These findings showed that intestinal dysbiosis leads to microbiota shifts, including metabolic disarrangements, inflammation, immunosuppression, and accumulation of uremic toxins, finally resulting in kidney failure.

Accumulating evidence has demonstrated that a bidirectional relationship existed in the host and gut microbiome in various kidney diseases. In this study, there are two issues that should be emphasized: Firstly, we totally agree with the important role of the microbiome in kidney diseases. Actually, as compared with intestinal dysbiosis, the imbalance between intestinal dysbiosis and liver is more important to renal function. The gastrointestinal tract mainly interacts with liver through the portal circulation (2). In enterohepatic circulation, chemicals that enter the digestive tract should be assimilated into portal venous blood by enterocytes, removed from blood by uptake into hepatocytes and secreted into the bile. Then these chemicals are deposited back into the intestinal lumen, which may be reabsorbed and reused by intestinal cells (3). As the intestine barrier is damaged, the liver is exposed to numerous toxic molecules and intestinal bacteria, thereby activating the hepatic innate immune system (4). The disruption of enterohepatic circulation results in the increase of toxins and renal injury. In addition, hepatorenal syndrome (HRS) can be caused by the imbalance between liver and kidney, which affects the kidney. Therefore, the role of hepatic function in gut-kidney axis should not be ignored.

The interaction between the intestinal function and renal function is bidirectional. Kidney diseases also affect the structure of gut microbiota and contributes to dysbiosis. As a result of gastrointestinal disorders associated with dysbiosis, the translocation of microbial compounds have been shown to have proinflammatory and nephrotoxic properties (5). In patients with chronic kidney disease (CKD), dysbiosis may lead to increased uremic toxin levels and then result in CKD progression (6). Hemodialysis is an effective treatment strategy for CKD. Hemodialysis cause the rupture of red blood cells, and then also transiently causes volume deficiency in the human body. In addition, strict control of the volume for patients with hemodialysis will cause loss of blood volume. Then it leads to intestinal hypoperfusion, mucosal ischemia and bacterial translocation (7). Intestinal inflammation and dysfunctional epithelial barrier accelerate systemic translocation of the bacterial-derived uremic toxins, thereby causing oxidative stress damage to the kidney (8).

In conclusion, we need to pay more attentions to the role of liver in the gut–kidney axis, and we should also focus on the effect of CKD and the changes of volume on gut–kidney axis during the hemodialysis. Further studies are greatly required.

## Author Contributions

All the authors conceived the idea and wrote the manuscript.

## Conflict of Interest

The authors declare that the research was conducted in the absence of any commercial or financial relationships that could be construed as a potential conflict of interest.
